# Cellulose Nanofiber Composite with Bimetallic Zeolite Imidazole Framework for Electrochemical Supercapacitors

**DOI:** 10.3390/nano11020395

**Published:** 2021-02-04

**Authors:** Hemraj M. Yadav, Jong Deok Park, Hyeong Cheol Kang, Jeonghun Kim, Jae-Joon Lee

**Affiliations:** 1 Research Center for Photoenergy Harvesting & Conversion Technology (*phct*), Department of Energy and Materials Engineering, Dongguk University, Seoul 04620, Korea; hemrajy@dongguk.edu (H.M.Y.); whdejr213@dongguk.edu (J.D.P.); knpitw1@dongguk.edu (H.C.K.); 2 Department of Chemistry, Kookmin University, 77 Jeongneung-ro, Seongbuk-gu, Seoul 02707, Korea

**Keywords:** zeolitic imidazole framework, cellulose nanofibers, composites, electrical double-layer supercapacitor

## Abstract

Cellulose nanofiber (CNF) and hybrid zeolite imidazole framework (HZ) are an emerging biomaterial and a porous carbonous material, respectively. The composite of these two materials could have versatile physiochemical characteristics. A cellulose nanofiber and cobalt-containing zeolite framework-based composite was prepared using an in-situ and eco-friendly chemical method followed by pyrolysis. The composite was comprised of cobalt nanoparticles decorated on highly graphitized N-doped nanoporous carbons (NPC) wrapped with carbon nanotubes (CNTs) produced from the direct carbonization of HZ. By varying the ratio of CNF in the composite, we determined the optimal concentration and characterized the derived samples using sophisticated techniques. Scanning electron microscopy (SEM), X-ray diffraction analysis (XRD), and X-ray photoelectron spectroscopy (XPS) confirmed the functionalization of CNF in the metallic cobalt-covered N-doped NPC wrapped with CNTs. The CNF–HZNPC composite electrodes show superior electrochemical performance, which is suitable for supercapacitor applications; its specific capacitance is 146 F/g at 1 A/g. Furthermore, the composite electrodes retain a cycling stability of about 90% over 2000 charge–discharge cycles at 10 A/g. The superior electrochemical properties of the cellulose make it a promising candidate for developing electrodes for energy storage applications.

## 1. Introduction

With rapid urbanization, the frontiers of energy harvesting and storage technologies have been expanding massively. Technologies such as solar devices, perovskite based materials, fuel cells, Li-ion batteries, and supercapacitors are being investigated and applied widely [1,2,3]. Supercapacitors have dominated the research in this field owing to their low cost, environmentally benign operation, long cycling life, high power, and energy density [4,5,6,7]. Supercapacitors, an essential group of electric energy storage systems, link the space between conventional capacitors and batteries [8]. Typically, supercapacitors are divided into two categories, namely, electrical double-layer capacitors (EDLCs) and pseudocapacitors, based on the charge storage mechanism and the active materials employed [9,10,11,12,13].

The characteristics of supercapacitors make it suitable for utilization in wearable electronics, compact devices, automobiles, medical devices, and energy harvesting techniques [14,15]. Many of these systems need eco-friendly substances as their active material. However, the use of a single material is insufficient to satisfy this requirement. Composite materials, which possess hybrid characteristics superior to their individual components, have the potential to meet these needs [16]. The development of eco-friendly hybrid nanocomposites has also become a prime principle of environmental sustainability. 

Cellulose-based polymers are abundant, soluble, cheap, and high-viscosity biopolymers used in the textile, paper, food, and drug industries. Cellulose nanofibers (CNFs) are an important class of biopolymers and a derivative of cellulose that is rich in carboxyl groups [17]. CNFs have attracted considerable interest given their high surface area, abundance, recyclability, low coefficient of thermal expansion, biodegradability, and superior mechanical and optical properties [18]. CNFs can create a 3D network that offers an electron transport route and reinforcing effects that improve energy transfer, owing to their high crystallinity, aspect ratio, and specific surface area [19]. Furthermore, several attempts have been performed to use CNF based components in electrochemical energy storage systems such as Li-ion or Li-S batteries [20,21,22,23]. 

A metal–organic framework (MOF) has the advantages of easy synthesis, well-developed pores, high surface area, and the use of diverse species [24,25]. Therefore, MOFs have been used for the synthesis of porous carbon [26,27,28]. The prepared carbons showed excellent properties such as a high surface area, good electrical conductivity, catalytic properties, and redox activity [29,30,31,32]. Given these properties, MOF-derived carbons have been used in various applications [33,34,35,36,37]. Recently, nanocomposites of biopolymers with MOF have attracted immense interest in scientific reactions and technological applications [38]. The combination of CNFs and carbonous materials could create unique nanocomposites with integrated advantages and versatile functionalities [8,18]. The composites of CNF with carbon nanostructures can have improved mechanical, electrical, and electrochemical properties. So far, very few studies have reported on the integration of MOF-derived carbon onto CNF. 

Nystrom et al. [39] reported the preparation of an aerogel supercapacitor of nanocellulose with carbon nanotubes (CNTs). Kuzmenko et al. [40] demonstrated the preparation of a highly conductive CNF-reduced graphene composite electrode with superb volumetric capacitance, energy density, and power density (up to 46 F/cm^3^, 1.46 Wh/L, and 1.09 kW/L, respectively). Zhou et al. [41] fabricated paper using conductive nanolayers of MOF on CNFs, which demonstrated excellent mechanical properties and a high gravimetric capacitance of 141.5 F/g at a current density of 0.075 A/g. 

Here, we present an eco-friendly composite of a CNF and MOF-derived carbon synthesized via a simple chemical method. The content of the CNF was also optimized to deliver superior electrochemical performance. The prepared composites were characterized by various sophisticated techniques. The ability of CNF to create a conductive pathway within the carbon structure in the composite makes it an optimal material for energy storage applications. 

## 2. Materials and Methods

### 2.1. Materials

2-methylimidazole (2-MIM), zinc acetate dihydrate, cobalt acetate tetrahydrate, and Nafion^®^ perfluorinated resin solution were procured from Sigma Aldrich Korea (Seoul, Korea). Potassium hydroxide and ethanol were purchased from DaeJung (Gyeonggi-do, Korea). CNF was prepared as described in a previous study [42]. Double-distilled water was used for all experiments. All materials were used as received. 

### 2.2. Synthesis of CNF–HZ Composites

The hybrid zeolite imidazole framework (HZ) powders were synthesized with a slight modification to a previously described method [43]. Typically, in order to develop cobalt nanoparticles and CNTs, the ratio of Co^2+^: Zn^2+^ was kept at 2:1. The appropriate amount of CNF solution was sonicated for 30 min in a 100 mL beaker of water. Next, cobalt acetate tetrahydrate (0.06 M) and zinc acetate dihydrate (0.03 M) were dissolved via magnetic stirring at room temperature. The aqueous solution of 2-MIM (0.7 M) was poured in the above mixture under vigorous stirring at 1000 rpm for 15 min. The mixture was kept for 24 h at room temperature. The cellulose nanofibers-hybrid zeolite imidazole framework (CNF–HZ) powder was collected by centrifuging at around 7000 rpm and washed with ethanol and water. Finally, the sample was dried at 60 °C under a vacuum furnace to get composites of the CNF (1, 3, and 5 wt.%) with HZ. The HZ powder was prepared as described above without the addition of the CNF. 

**Figure 1:**
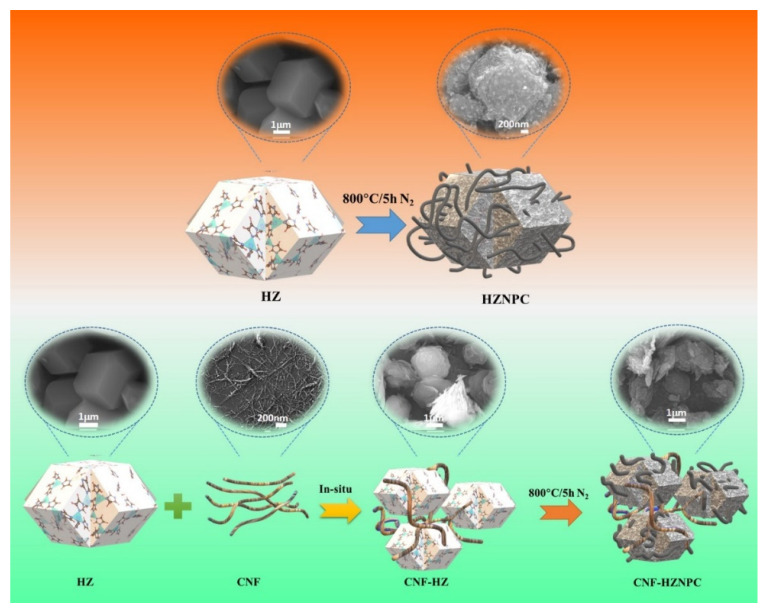
Schematic illustration of preparation of hybrid zeolite imidazole framework nanoporous carbon (HZNPC)and its composite with cellulose nanofibers (CNF).

Initially, nitrogen was passed over the samples for 30 min in a tube furnace at room temperature. Then the samples were pyrolyzed at 800 °C for 5 h under a continuous nitrogen gas flow in the tube furnace to form hybrid zeolite imidazole framework nanoporous carbon (HZNPC) and CNF–HZNPC (1, 3, and 5 wt.%). A heating rate of 2 °C/min was applied for the carbonization process. The schematic for the preparation of the samples is shown in Figure 1. For comparison, a 3% CNF–HZNPC composite was prepared by physically mixing HZNPC and CNF. The photograph of powder sample is shown in Figure S1a.

### 2.3. Characterization

The synthesized HZ and the nanocomposites of CNF–HZ were characterized using different microscopic and spectroscopic techniques. XRD patterns were acquired on a diffractometer (Bruker D8 Advance, Billerica, Germany) with a Cu target (40 kV, 40 mA) from 10° to 80° at a scan rate of 3° min^−1^ with a step size of 0.02°. The surface morphologies of the samples were observed using SEM (JEM-3010, JEOL Ltd., Tokyo, Japan) 15 kV in a high vacuum. N_2_ adsorption–desorption measurement was carried out using a Micromeritics ASAP 2460 (Norcross, GA, USA) analyzer at −196 °C. X-ray photoelectron spectroscopy (XPS) analysis was conducted on a Veresprobe II (ULVAC-PHI, Kanagawa, Japan) equipped with a monochromatic Al-Kα X-ray source. 

### 2.4. Electrochemical Study

Electrochemical measurements were performed using a VersaSTAT 3 potentiostat. A platinum foil and Hg/HgO were used as the reference electrode and counter electrode, respectively. A glassy carbon substrate (25 × 25 mm^2^) was used as the working electrode. 1 mg powder was mixed with 100 μL of ethanolic solution of Nafion^®^ (0.2 wt.%) binder using an ultrasonication bath for 30 min. The resultant colloidal slurry was coated on a glassy carbon electrode (GCE). Cyclic voltammetry (CV) analysis was conducted in a 1 M KOH aqueous solution by potential cycling between −0.8 and 0.4 V (vs. Hg/HgO) at scan rates of 5 to 300 mV/s. Galvanostatic charge–discharge (GCD) analysis was performed by scanning the potential from −0.8 to 0.4 V (vs. Hg/HgO) at current densities from 1 to 10 A/g in 1 M KOH. Each electrode was fabricated and electrochemically tested at room temperature for four times. 

## 3. Results

**Figure 2:**
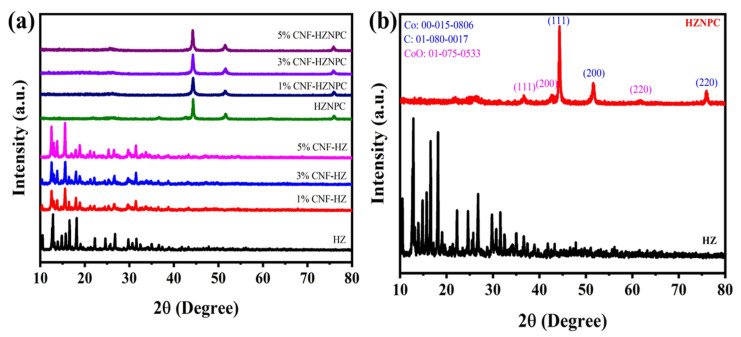
XRD patterns of (**a**) all samples(**b**) HZ and HZNPC.

The XRD patterns of all samples are shown in Figure 2a. Figure 2b shows the XRD pattern of HZ and HZNPC. The XRD patterns of the prepared HZ and HZNPC matches exactly with the XRD pattern that was reported previously [12,43]. The intensity of the characteristic peak of the CNF at around 15.6° increased with the increase in CNF in the CNF–HZ composite (Figure 2a). This increase in the XRD peak intensity of CNF implies that crystallinity has been increased and its homogenous distribution on the HZ. The presence of broad peaks around 25.5° and 42.64° indicates an alignment of carbon layer planes. The XRD patterns of the carbonized sample appearing at 36.62°, 42.64°, and 61.94° are assigned for CoO while the peaks at 44.32°, 51.6°, and 75.96° match with the metallic Co (00-015-0806) [43,44]. The most intense peak at 44.32° also matches with the carbon (01-080-0017). The of Zn particles was decomposed at high temperature as its melting point is lower (~600 °C) than carbonization temperature (800 °C) [45]. 

**Figure 3:**
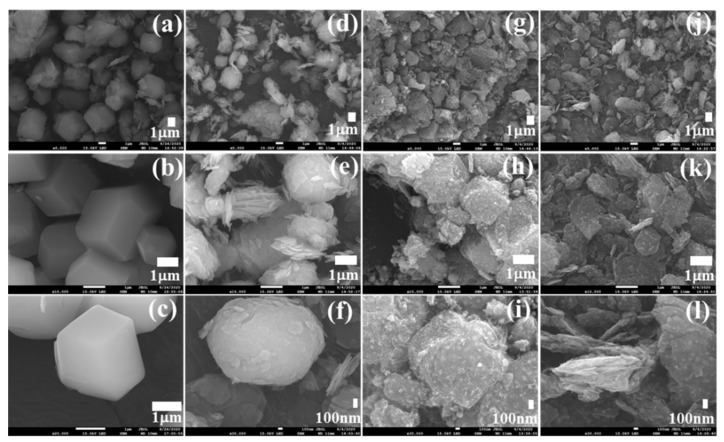
SEM images of (**a**–**c**) HZ, (**d**–**f**) 3% CNF-HZ, (**g**–**i**) HZNPC, (**j**–**l**) 3% CNF-HZNPC.

The morphological features of the samples were observed using FESEM analysis. FESEM images of HZ and composite HZ before and after carbonization are shown in Figure 3. The FESEM image for CNF is shown in Figure S1b. A nanofiber network with a highly interconnected sheet-like structure was observed for CNF. The length of nanofiber was a few nanometers, and the diameter was less than 20 nm. FESEM images of as-prepared HZ are shown in Figure 3a–c. A rhombic dodecahedral structure can be observed for the HZ before carbonization. After carbonization, the polyhedral carbon structure derived from ZIF is destroyed during the formation of metallic cobalt and some cobalt oxide. As reported previously, the carbonization with a lower heating rate of 2 °C/min was essential to develop the CNTs (Figure 3g–i) [43]. The structure of the 3% CNF–HZ is like the structure of HZ, while after incorporation of CNF and carbonization, most of the rhombic dodecahedral portion is destroyed (Figure 3j–l). It has been reported that the catalytic active Co metal particles present on the carbon matrix can enhance the degree of graphitization, resulting in the growth of CNTs [45]. The existence of CNTs and CNF enhances the electrical conductivity of the HZ composite.

N_2_ adsorption-desorption isotherm of HZNPC and 3% CNF-HZNPC is shown in Figure S1c. The surface area of 3% CNF-HZNPC was 316.97 m^2^/g, which is higher than HZNPC (306.67 m^2^/g). The incorporation of CNF showed slightly increased surface area of the composite, which can provide advantage for supercapacitor application. 

**Figure 4:**
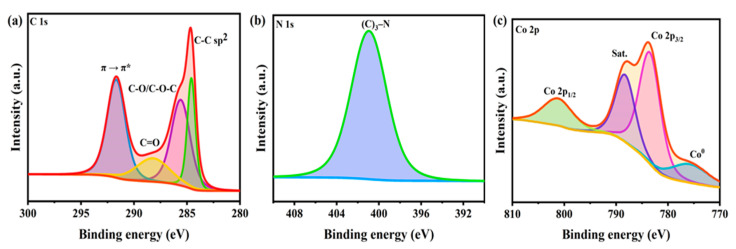
XPS spectra of 3% CNF-HZNPC. (**a**) C 1sa, (**b**) N 1s and (**c**) Co 2p.

X-ray photoelectron spectroscopy (XPS) measurement was used to verify the surface chemical composition of the 3% CNF–HZNPC (Figure 4). The deconvolution of C1s peaks is shown in Figure 4a. The peak appeared at 284.58 and 287.99 for C–C sp^3^/C=C sp^2^ and C=O, respectively. The broad peak at 285.58 is assigned to C–O and C–O–C groups [46]. The π → π* shake-up satellite peak at 291.2 eV is characteristic of an aromatic or conjugated system, confirming the restoration of the sp^2^-hybridized carbon network [47]. The N1s peaks at 400.2 eV confirm that nitrogen is present in the form of (C)3–N (sp^3^) (Figure 4b) [48]. These results reveal that the carbon is doped with nitrogen. The 2p^3/2^ feature located at 783.49 eV and the associated 2p^1/2^ peak at 800.20 eV exhibit an energy splitting (ΔE = 16.71 eV), which is also consistent with a Co^2+^ state (Figure 4c). The satellite line for the high spin Co^2+^ is at 788.26 eV [49]. The peak at 775.78 eV appeared for Co^0^, confirming the existence of metallic Co on the surface of the carbonized sample. 

**Figure 5:**
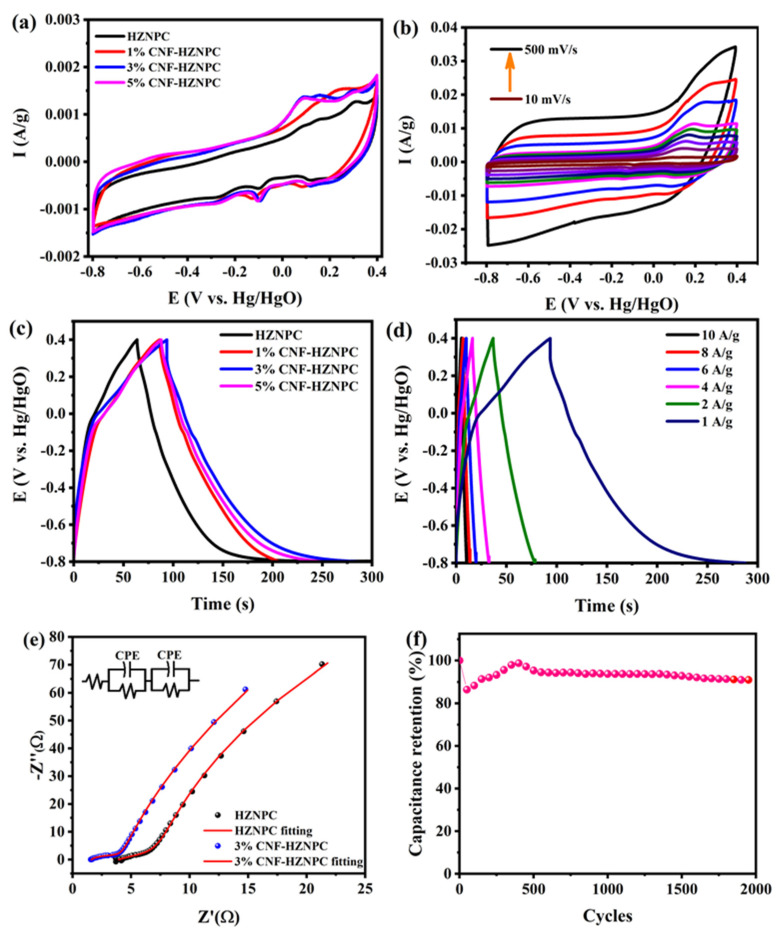
(**a**) CV of HZNPC and CNF-HZNPC composites, (**b**) CV of 3% CNF-HZNPC at different scan rate, (**c**) GCD of HZNPC and CNF-HZNPC composites at current density of 1 A/g, (**d**) GCD of 3% CNF-HZNPC at different current densities, (**e**) EIS of HZNPC and 3% CNF-HZNPC, (**f**) cycling performance of 3% CNF-HZNPC at 10 A/g.

To compare the electrochemical properties of HZNPC and CNF–HZNPC, CV and GCD tests were conducted using a three-electrode system. Figure 5a shows the CV curves of the HZNPC and CNF–HZNPC at a scan rate of 50 mV/s in the voltage range −0.8 to +0.4 V in a 1 M KOH solution. All the samples retained almost rectangular CV profiles, demonstrating desired electric double-layer capacitance (EDLC) behavior. The small humps detected in the CV curves as a result of faradaic pseudo-capacitance arise from the existence of the metallic cobalt species. The electroactivity of the CNF-modified HZNPC was improved compared to the unmodified electrode. The CV area increased with the increase in CNF in the composite, from 1% to 3%. The CV area for 5% is almost similar to the 3% CNF-containing sample, revealing that beyond 3% there is a negligible increment in the CV area of the composite. The CV curves of 3% CNF–HZNPC at scan rates of 10, 30, 50, 70, 90, 110, 200, 300, and 500 mV/s in 1 M KOH are depicted in Figure 5b. The CV curves for 3% CNF–HZNPC exhibited a rectangular shape with the increase in the scan rates, which indicates excellent rate capability, characteristics of reversible and EDLC behavior [50]. 

The GCD curves of all samples were obtained at a current density of 1 A/g in the potential range of −0.8 to +0.4 V in 1 M KOH solution (Figure 5c). The symmetrical and slightly distorted GCD curves imply superior capacitive behavior. The specific capacitance (Cs) is estimated from the equation: Cs=(I × ∆t)/∆V × m), where I is the charge–discharge current (A), ∆t is the discharge time, ∆V is the potential window (V), and  is the weight of the active material (g). The Cs values of HZNPC, 1% CNF–HZNPC, 3% CNF–HZNPC, and 5% CNF–HZNPC were estimated to be 115, 130, 146, and 101 F/g at 1 A/g, respectively. Among all CNF-modified samples, the 3% sample exhibited higher specific capacitance than other samples. Furthermore, the specific capacitance values of 3% CNF–HZNPC were found to be 146, 69.35, 55.83, 50, 45.66, and 43.33 F/g at an applied current density of 10, 8, 6, 4, 2, and 1 A/g, respectively. The specific capacitance was enhanced to 27% because of the incorporation of CNF compared to the initial capacitance of HZNPC. The specific capacitance of HZNPC and 3% CNF-HZNPC with different current density is shown in Figure S2c. This improvement in the specific capacitance can be attributed to the introduction of CNF improving the electrical conductivity, wettability, and specific surface area of the composites, which can promote electron transfer and provide more electrochemically active sites [10,19,51]. The electrochemical performance of 3% CNF–HZNPC was compared with the physical mixture of HZNPC and CNF (Figure S2a,b). The performance of the physical mixture was lower (106 F/g at 1 A/g) than that of the sample prepared via in situ method, which indicated less interaction of CNF with HZNPC after physical mixing. Electrochemical impedance spectroscopy analysis was conducted. Plots of the HZNPC and 3% CNF–HZNPC electrodes are shown in Figure 5e. The 3% CNF–HZNPC electrode presents a more vertical line in the low-frequency region compared with HZNPC, indicating a better capacitive behavior [52]. Furthermore, the solution resistance (Rs), charge transfer impedance at the electrolyte/electrode interface (R_ct-1_ and R_ct-2_), and goodness-of-fit (χ^2^) values are 3.9 Ω, 4.03 Ω, 52.48 Ω, and 2.93 × 10^−3^ for HZNPC and 1.6 Ω, 2.52 Ω, 8.1 Ω, and 4.62 × 10^−4^ for 3% CNF-HZNPC, respectively. The 3% CNF–HZNPC showed much lower resistance than HZNPC. The nanoporous structure and high surface area of the composite could promote faster ion diffusion resulting in low R_ct_ values [53,54]. Therefore, the enhanced the electrochemical performance of 3% CNF–HZNPC can be attributed to its excellent impedance characteristics and good electrical conductivity. 

Cycling stability is an important parameter to validate the practicability of the electrode material. The electrochemical stability of the 1, 3, and 5% CNF–HZNPC was also investigated. The 3% CNF–HZNPC electrode maintained a capacitance retention of 90% of its initial capacitance after 2000 cycles at a current density of 10 A/g in a 1 M KOH solution (Figure 5f). The higher value of capacitance retention reveal that the electrode might be stable for long cycles. The cycling performance of 1 and 5% CNF-HZNPC was decreased to about 11 and 18%, respectively (Figure S2d). The excellent cycling stability can be attributed to the 3D network of CNF and CNTs of HZ. The result demonstrates that 3% CNF–HZNPC is a promising electrochemical electrode material with excellent stability. 

## 4. Conclusions

An environmental-friendly composite of CNF–HZNPC has been prepared via pyrolysis using a bimetallic hybrid zeolite imidazole framework. The optimum concentration of CNF in the composite electrode significantly enhanced electrochemical performance of the CNF–HZNPC composite. The specific capacitance of 3% CNF–HZNPC is about 146 F/g at 1 A/g, with an excellent cycling stability of 90% after 2000 cycles and low charge-transfer resistance. Such improved electrochemical performance could be attributed to the synergetic effect of an optimum concentration of CNF, the EDLC, and the optimal pseudocapacitance nature of the composite. The present work describes a strategy for improving the electrochemical performance of ZIF-derived carbons using biodegradable CNF for energy storage applications.

## Data Availability

The data presented in this study are available on request from the corresponding author. The data are not publicly available due to privacy. Data is contained within the article or supplementary material.

## Supplementary Materials

The following are available online at https://www.mdpi.com/2079-4991/11/2/395/s1, Figure S1: (a) Photograph of powder samples (b) FESEM image of CNF (Inset: High magnification) (c) N_2_ adsorption-desorption isotherm of HZNPC and 3% CNF-HZNPC. Figure S2: (a,b) CV and GCD of 3% CNF-HZNPC physical mixture at different scan rate and current density values, respectively. (c) Current density vs. specific capacitance for HZNPC and 3% CNF-HZNPC. (d) Cycling performance of 1% CNF-HZNPC and 5% CNF-HZNPC at 10 A/g.
