# Supplementary material for: Cellulose Nanofiber Composite with Bimetallic Zeolite Imidazole Framework for Electrochemical Supercapacitors
Source: Nanomaterials (Basel). 2021 Feb 4;11(2):395. doi: 10.3390/nano11020395 (PMC7913791; doi:10.3390/nano11020395)
Supplement: Supplementary file 1 [file nanomaterials-11-00395-s001.pdf]

nanomaterials

MDPI

Supplementary Materials

Cellulose Nanofiber Composite with Bimetallic Zeolite Imidazole Framework for Electrochemical Supercapacitors

Hemraj M. Yadav [1], Jong Deok Park [1], Hyeong Cheol Kang [1], Jeonghun Kim [2,*] and Jae-Joon Lee [1,*]

1 Research Center for Photoenergy Harvesting & Conversion Technology (*phct*), Department of Energy and Materials Engineering, Dongguk University, Seoul 04620, Korea; hemrajy@dongguk.edu (H.M.Y.); whdejr213@dongguk.edu (J.D.P.); knpitw1@dongguk.edu (H.C.K.)

2 Department of Chemistry, Kookmin University, 77 Jeongneung-ro, Seongbuk-gu, Seoul 02707, Korea

* Correspondence: jeonghunkim@kookmin.ac.kr (J.K.); jjlee@dongguk.edu (J.-J.L.); Tel: +82-10-4659-8255 or +82-2-2260-4979/8513 (J.-J.L.)

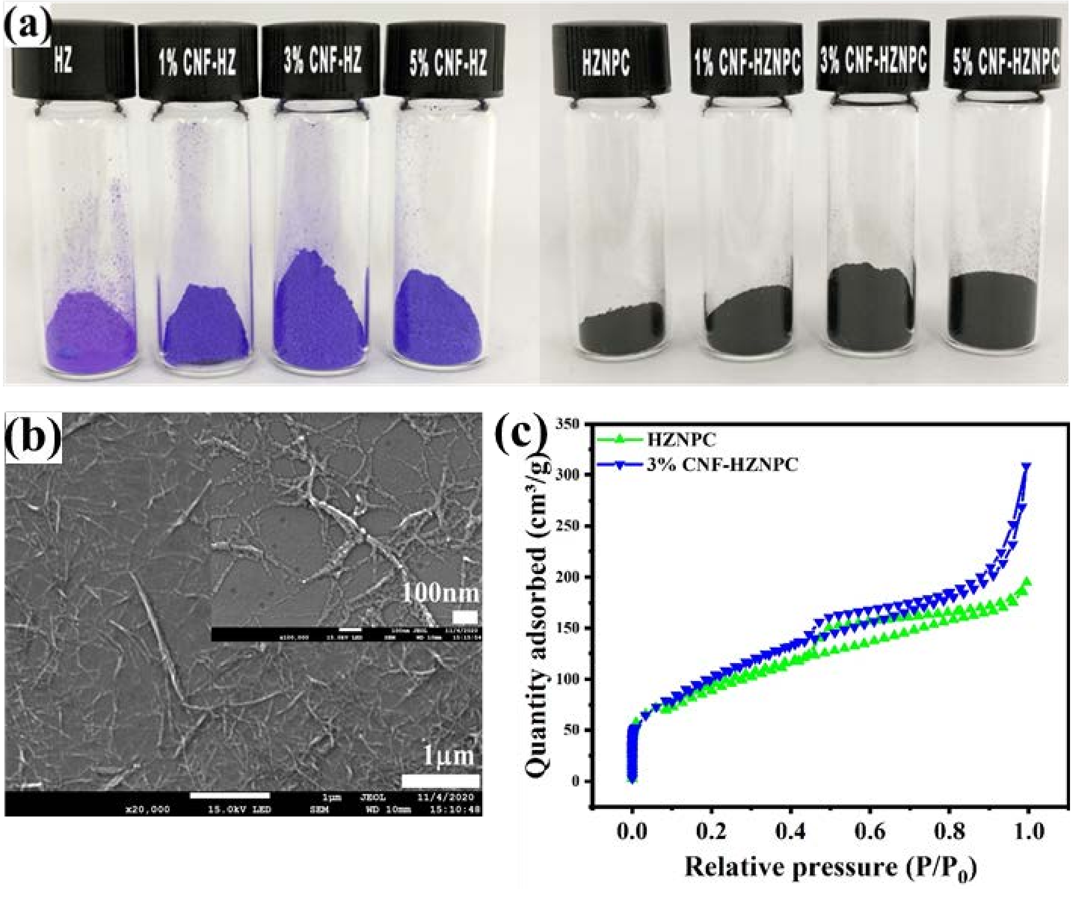

Figure S1. (**a**) Photograph of powder samples (**b**) FESEM image of CNF (Inset: High magnification) (**c**) N_2 adsorption-desorption isotherm of HZNPC and 3% CNF-HZNPC.

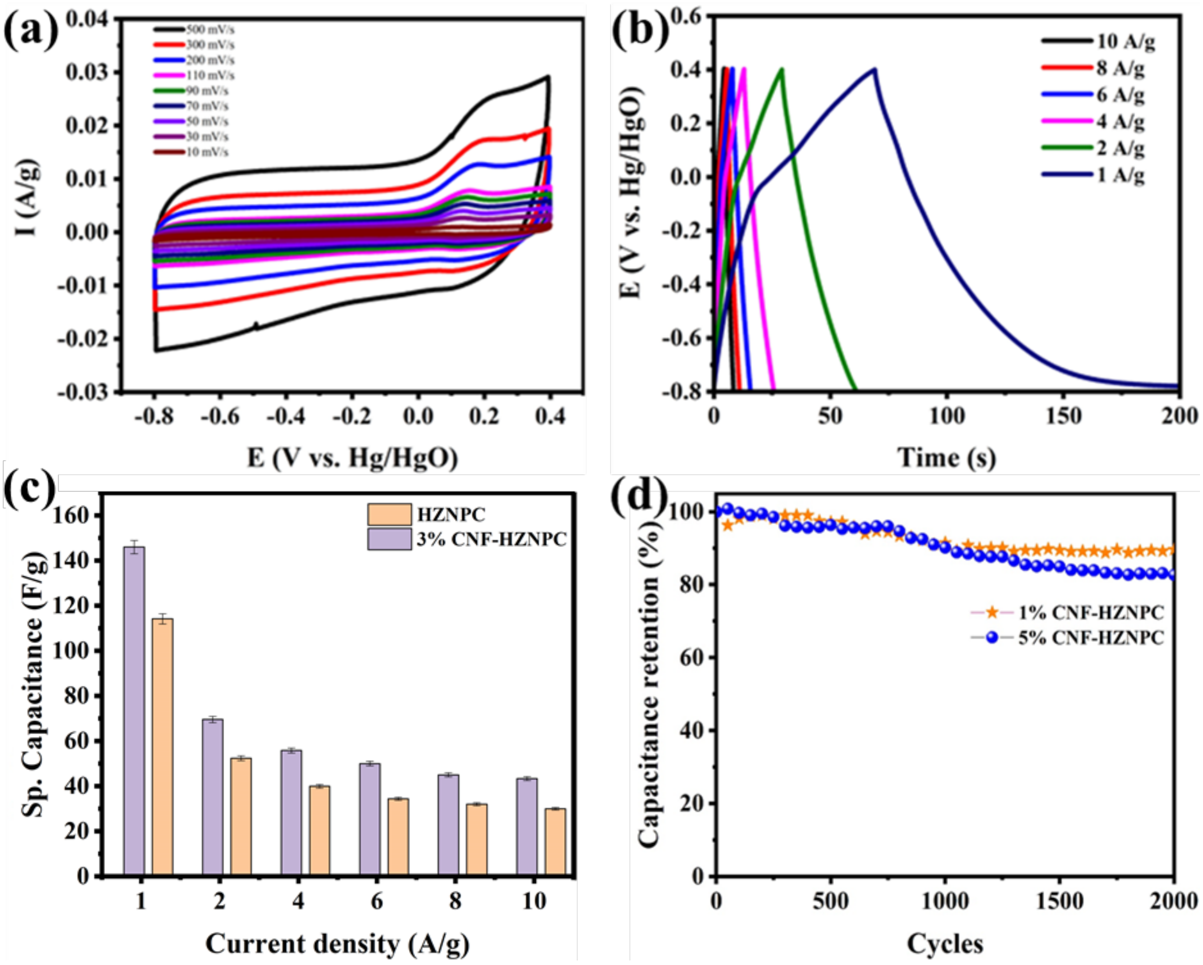

Figure S2. (**a-b**) CV and GCD of 3% CNF-HZNPC physical mixture at different scan rate and current density values, respectively. (**c**) Current density vs specific capacitance for HZNPC and 3% CNF-HZNPC. (**d**) Cycling performance of 1% CNF-HZNPC and 5% CNF-HZNPC at 10 A/g.
